# SNHG1 promotes cell proliferation by acting as a sponge of miR-145 in colorectal cancer

**DOI:** 10.18632/oncotarget.23255

**Published:** 2017-12-14

**Authors:** Tian Tian, Ran Qiu, Xia Qiu

**Affiliations:** ^1^ Department of Neurology, The First Affiliated Hospital of Zhengzhou University, Zhengzhou, People's Republic of China; ^2^ Wuhan Institute of Bioengineering, Wuhan, People's Republic of China; ^3^ Department of Medicine, Shangqiu Medical School, Shangqiu, People's Republic of China

**Keywords:** SNHG1, miR-145, colorectal cancer, ncRNA, lncRNA

## Abstract

ncRNAs are important regulatory molecules and involve in many physiological cellular processes. Small nucleolar RNA host gene 1 (SNHG1) is a host to 8 snoRNAs and is located in 11q12.3 region of the chromosome. It has been reported to be involved in several cancers. However, the role of SNHG1 in the tumorigenesis of colorectal cancer is still unknown. In this study, SNHG1 was upregulated in colorectal cancers, and SNHG1 expression was correlated with advanced colorectal cancer stage and tumor recurrence. We found that SNHG1 promoted cell proliferation by acting as a sponge of miR-145, a well known tumor suppressor of colorectal cancer. Furthermore, the survival analysis indicated that colorectal cancer patients with higher expression of SNHG1 had a worse prognosis. These findings suggested that SNHG1 may act as a potential therapeutic target for the treatment of colorectal cancer.

## INTRODUCTION

Colorectal cancer is the most prevalent cancer and one of the leading causes of cancer death worldwide [1, 2]. Although recent advances in the diagnosis and management of colorectal cancer, the prognosis of patients with advanced stage of colorectal cancer is still poor [3–6]. Therefore, it is urgent to clarify the molecular pathogenesis of the cancer progression, and which might contribute significantly to the development of new diagnostic strategies and potential therapeutic targets.

Recently, large numbers of RNA transcripts which lack protein-coding potential have been found, and which were referred to as non-coding RNAs (ncRNAs)[7–10]. ncRNAs are important regulatory molecules and involve in many physiological cellular processes [8, 11–13]. It's found that deregulated expression of ncRNAs have an important roles in the process of tumorigenesis [9, 14–18]. Small nucleolar RNAs (snoRNAs) is another large class of ncRNAs [19, 20]. Recent studies indicate that they could affect cell proliferation, transformation and tumorigenesis in a variety of human cancers [21–23]. Small nucleolar RNA host gene 1 (SNHG1) is a host to 8 snoRNAs and is located in 11q12.3 region of the chromosome [24, 25]. It has been reported to be involved in several cancers. SNHG1 is upregulated in hepatocellular carcinoma, and the upregulated expression of SNHG1 predicts a poor prognosis [26, 27]. Moreover, it's reported that SNHG1 may contribute to the aggravation of hepatocellular carcinoma through the inhibition of miR-195 [26]. In lung cancer, it's found that SNHG1 could contribute to cancer progression via inhibition of miR-101-3p and activation of Wnt/β-catenin signaling pathway [28]. In prostate cancer, SNHG1 negatively regulates miR-199a-3p to enhance CDK7 expression and promote cell proliferation [29]. In esophageal cancer, SNHG1 was reported to act as a non-degradable sponge for miR-338 to promote esophageal carcinoma cell growth [30]. However, the role of SNHG1 in the tumorigenesis of colorectal cancer is still unkown.

In this study, SNHG1 was upregulated in colorectal cancers, and SNHG1 expression was correlated with advanced colorectal cancer stage and tumor recurrence. We found that SNHG1 promoted cell proliferation by acting as a sponge of miR-145, a well known tumor suppressor of colorectal cancer. Furthermore, the survival analysis indicated that colorectal cancer patients with higher expression of SNHG1 had a worse prognosis.

## RESULTS

### SNHG1 is upregulated in colorectal cancers

**Figure 1:**
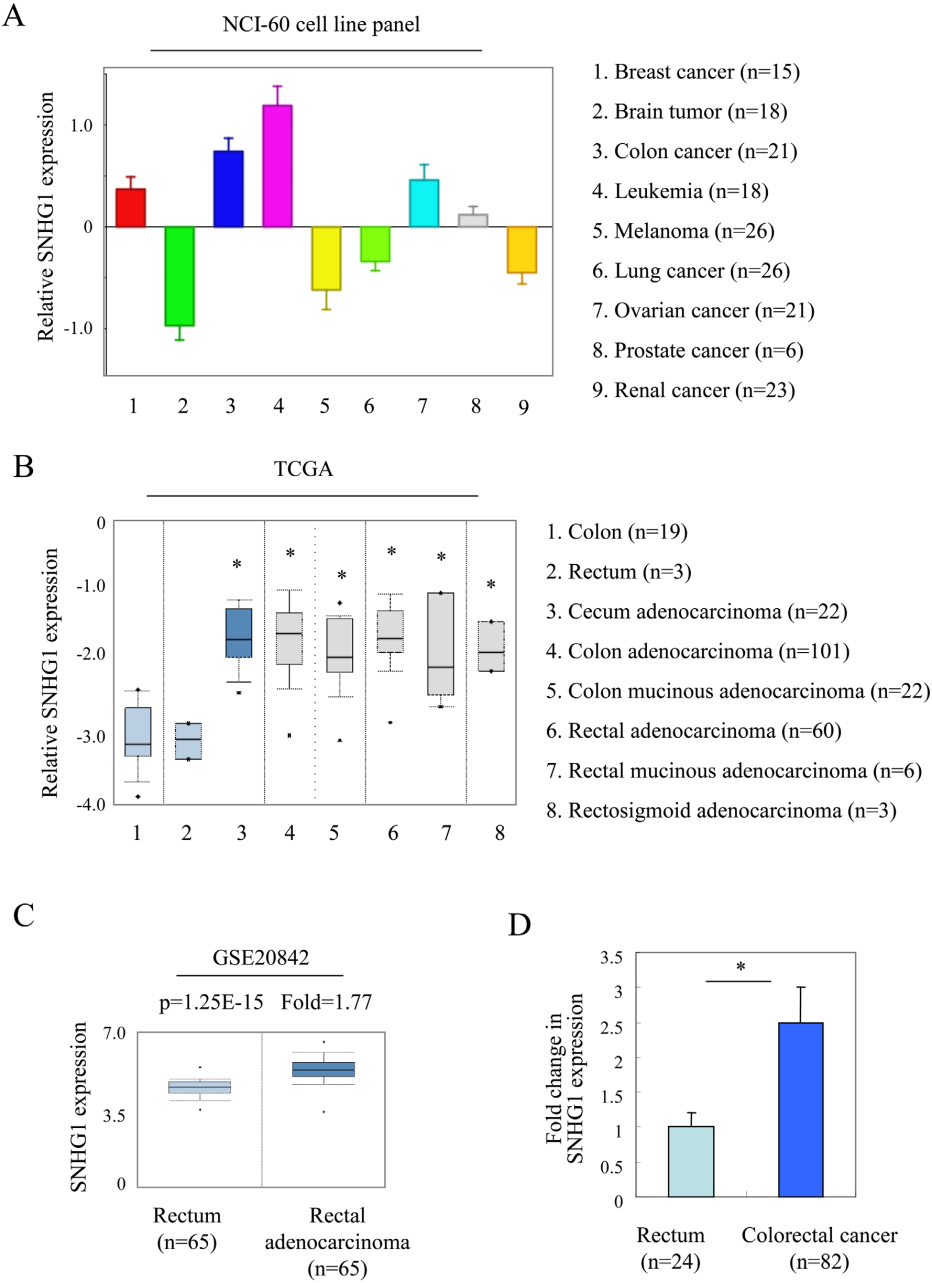
SNHG1 is upregulated in colorectal cancers **(A)** mRNA expression of SNHG1 in various cancers was determined by the analyzing the NCI-60 cell line microarray dataset in R2 microarray analysis and visualization platform. **(B, C)** The expression of SNHG1 is upregulated in colorectal cancer when compared with non-tumor colorectal tissues. TCGA colorectal cancer (B) and GSE20842 (C) datasets were analyzed. All data, including fold change and *p*-values, were calculated from ONCOMINE (www.oncomine.org). **(D)** The expression of SNHG1 in 82 colorectal cancer tissues and 24 non-tumor rectum tissues were examined by real-time PCR. Bars, s.e.m.; ^*^, *p*<0.05.

We examined the expression of SNHG1 in colorectal cancer. First, mRNA expression of SNHG1 in various cancers was determined by the analyzing the NCI-60 cell line microarray dataset in R2 microarray analysis and visualization platform. Across a large collection of different cancer types, the expression of SNHG1 was abundant in colorectal cancer cell lines (Figure 1A). We also tested the expression of SNHG1 by querying the public available ONCOMINE database (www.oncomine.org) [31] in colorectal cancers. Two colorectal cancer microarray expression datasets (TCGA and GSE20842) were analyzed. The TCGA dataset showed that the expression of SNHG1 mRNA is significantly higher in different subtypes of colorectal cancer than that in the non-tumor colorectal tissues (colon or rectum) in these microarray studies (^*^, *p<*0.05, Figure 1B). The result of GSE20842 demonstrated that SNHG1 expression was 1.77-fold higher in cancer tissues as compared with the non-tumor rectum tissues (^*^, *p*=1.25E-15, Figure 1C). Moreover, we examined the expression of SNHG1 in 82 colorectal cancer tissues and 24 non-tumor rectum tissues through quantitative real-time PCR. As shown in Figure 1D, the expression of SNHG1 was 2.5-fold higher in colorectal cancer tissue than non-tumor rectum tissues (^*^, *p<*0.05).

Next, we analyzed the correlation between the expression of SNHG1 and their clinical characteristics in colorectal cancer (Table 1). There was no significant association between SNHG1 expression and age, gender, tumor location, tumor grade and distant metastases, except for tumor stage and tumor recurrence (Table 1).

**Table 1:** The relationship between SNHG1 expression and clinical characteristics of patients with colorectal cancer

Characteristics	SNHG1 expression		p value
	Low(n=41)	High(n=41)	
Age (years)			1.000
<60	11	10	
≥60	30	31	
Gender			0.818
Male	16	14	
Female	25	27	
Tumor location			0.755
Proximal colon	18	17	
Distal colon	12	15	
Rectum	11	9	
Tumor grade			0.311
G1/G2	33	28	
G3/G4	8	13	
Stage			0.007
I / II	31	18	
III / IV	10	23	
Distant metastases			0.141
Yes	4	10	
No	37	31	
Recurrence			0.032
Yes	8	18	
No	33	23	

### SNHG1 promotes tumor growth in colorectal cancer

**Figure 2:**
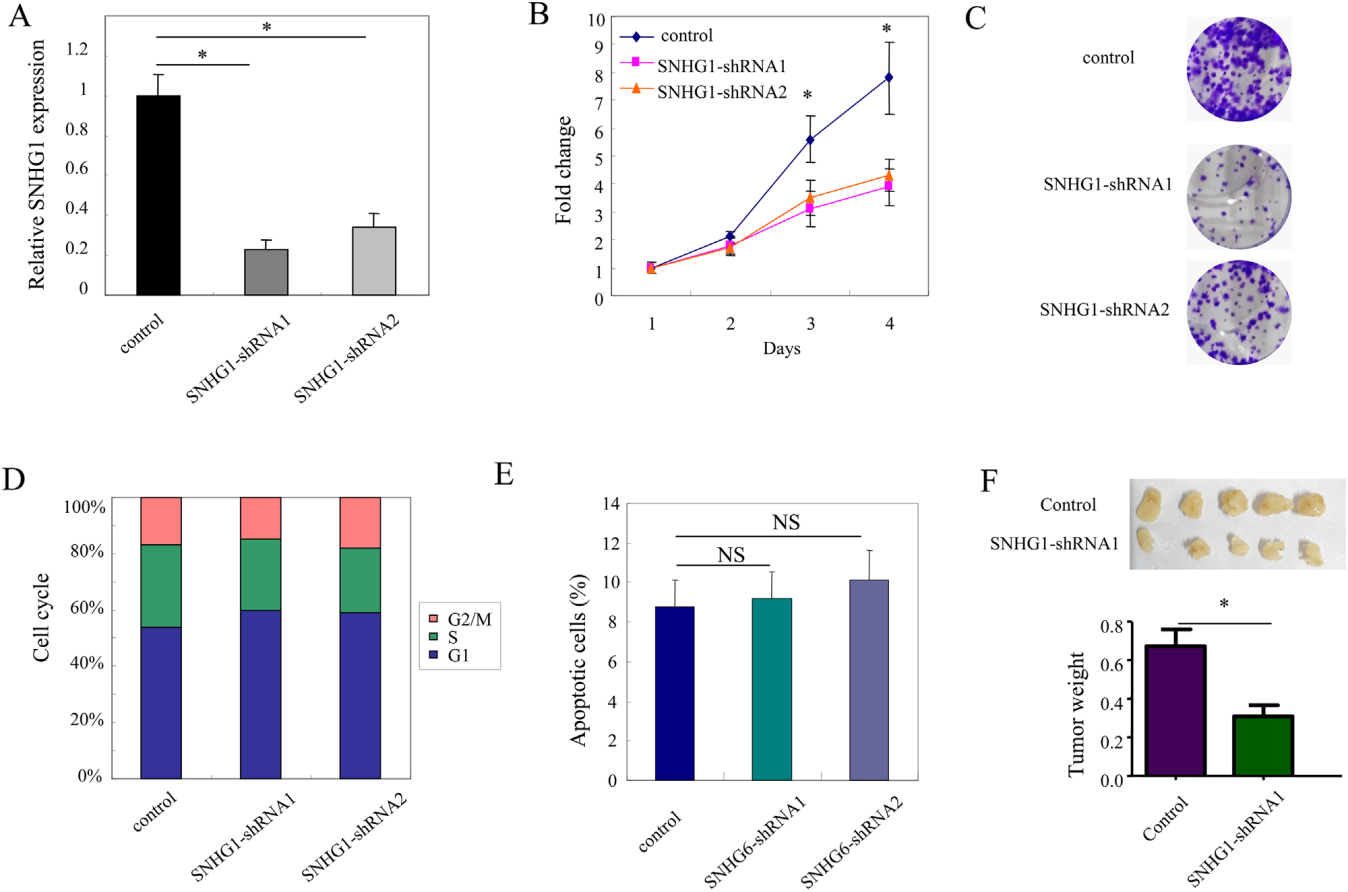
SNHG1 promotes tumor growth in colorectal cancer **(A)** SNHG1 expression was examined after knockdown of SNHG1 in LOVO cells. **(B-E)** The effect of SNHG1 on cell proliferation and cell death by performing CCK-8 cell growth assay (B), colony formation assay (C), cell cycle analysis (D) of and Annexin/PI staining in LOVO cells stably transfected either with SNHG1 shRNAs (SNHG1- shRNA1 and SNHG1- shRNA2). **(F)** *In vivo* in xenograft tumor model. LOVO cells with stable expression of either with SNHG1 shRNA1 or scramble control was subcutaneously injected into three groups of nude mice. Representative subcutaneous tumor xenografts and the weight of the tumors were examined. All experiments were performed in triplicate; bars, s.e.m.; ^*^, *p*<0.05; NS, not significant.

We next examined the effect of SNHG1 on the cell proliferation to determine the functional role of SNHG1 in of LOVO cells, a colorectal cancer cell line. We established LOVO cell line with knockdown of the endogenous expression of SNHG1 by lentiviral transduction. The quantitative real-time PCR results showed that SNHG1 expression was dramatically suppressed by SNHG1 specific shRNAs, SNHG1-shRNA1 and SNHG1-shRNA2 (^*^, *p<*0.05). Then, we examined the effect of SNHG1 on cell proliferation through CCK-8 cell growth assay and colony formation assay. As shown in Figure 2B, the cell growth assay demonstrated that knockdown of endogenous SNHG1 significantly suppressed cell growth in LOVO cells (^*^, *p<*0.05; Figure 2B). Consistently, the numbers of colonies of SNHG1-shRNA1 and SNHG1-shRNA2 group were much less than the vector control group (^*^, *p<*0.05, Figure 2C). We then assessed whether SNHG1 affected the cell cycle progression in LOVO cells. The results showed that knockdown of SNHG1 didn't change the cell cycle progression in LOVO cells (Figure 2D). Furthermore, the effect of SNHG1 on cell death was assessed by Annexin V/PI staining assay. However, no significant changes in cell death were observed after knockdown of endogenous SNHG1 expression in colorectal cancer cells (Figure 2E).

Furthermore, to validate these above findings, we performed xenograft tumor assay *in vivo*. Cells with stable expression of SNHG1-shRNA1 or vector control were subcutaneously injected into nude mice (n=5 in each group). Four weeks later, the mice were sacrificed and the tumors derived from each group were collected. As shown in Figure 2F, the average tumor weight of vector control and SNHG1-shRNA1 group were (0.71 ± 0.06) g and (0.35 ± 0.03) g, respectively (^*^, *p<*0.05). In summary, these results suggested that SNHG1 promoted tumor growth of colorectal cancer.

### SNHG1 functions as a sponge of miR-145 in colorectal cancer cells

**Figure 3:**
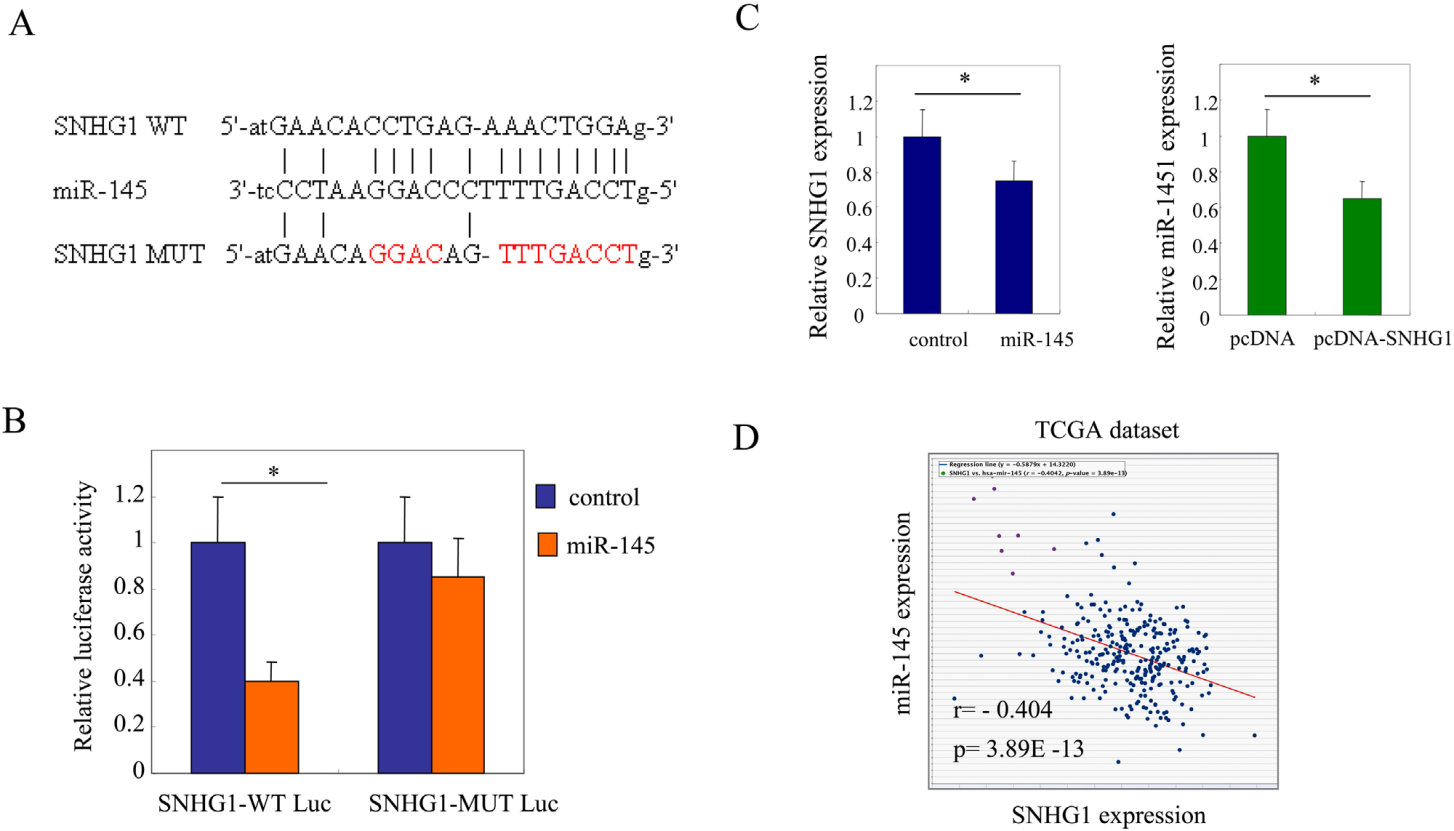
SNHG1 functions as a sponge of miR-145 in colorectal cancer cells **(A)** miRNA target sites prediction analysis was performed by the online software starBase v2.0. SNHG1 was predicted containing one binding sites to miR-145. **(B)** Luciferase reporter constructs containing wild-type binding site (SNHG1-WT Luc) or mutated binding site (SNHG1-MUT Luc) were transfected in LOVO cells in the presence of miR-145 or vector control for 24 h. Luciferase activity was determined. **(C)** SNHG1 expression after miR-145 overexpression in LOVO cells (left panel). Endogenous expression levels of miR-145 after SNHG1 overexpression in LOVO cells (right panel). **(D)** Co-expression of SNHG1 and miR-145 were analyzed through querying open database ChIPBase v2.0 in TCGA colorectal cancer datasets (r: -0.404, *p*=3.89E-13).

It's well known that lncRNAs act as sponges for miRNAs to regulate the downstream mRNA degradation and translation [32, 33]. Therefore, we hypothesized that SNHG1 might also work as a sponge to modulate certain miRNAs to promote cell proliferation in colorectal cancer cells. To analyze the potential miRNA targets of SNHG1, miRNA target sites prediction analysis was performed by the online software starBase v2.0. Considering the well known functions of miR-145 in colorectal cancer, it was selected for further study. The result showed one binding sequences in the SNHG1 transcripts were found pairing with miR-145 (Figure 3A). We then constructed luciferase reporter vectors containing the SNHG1 wild type (WT) or mutations at the putative miR-145 binding sites (MUT) (Figure 3A). LOVO cells were transfected with miR-145 in combination with either SNHG1 wild type (SNHG1-WT Luc) or SNHG1 mutated (SNHG1-MUT Luc) luciferase reporter vectors. As a result, overexpression of miR-145 reduced significantly the luciferase activity of the wild type SNHG1 reporter (SNHG1-WT Luc) but not the mutated SNHG1 reporter (SNHG1-MUT Luc) (^*^, *p<*0.05, Figure 3B). It indicated that SNHG1 might bind miR-145 in colorectal cancer cells.

Furthermore, the quantitative real-time PCR analysis showed overexpression of miR-145 led to a marked decrease of SNHG1 expression. Reciprocally, upregulation of SNHG1 repressed the expression of miR-145 in colorectal cancer cells (^*^, *p<*0.05, Figure 3C). The results indicated that endogenous expression levels of SNHG1 and miR-145 were negatively correlated with each other in colorectal cancer cells. Then, co-expression of SNHG1 and miR-145 were analyzed through querying open database ChIPBase v2.0 in TCGA colorectal cancer datasets. The results showed that SNHG1 and miR-145 was negatively correlated in colorectal cancers (r: -0.404, *p*=3.89E-13; Figure 3D).

### Target genes of miR-145 were modulated by SNHG1 in colorectal cancer cells

**Figure 4:**
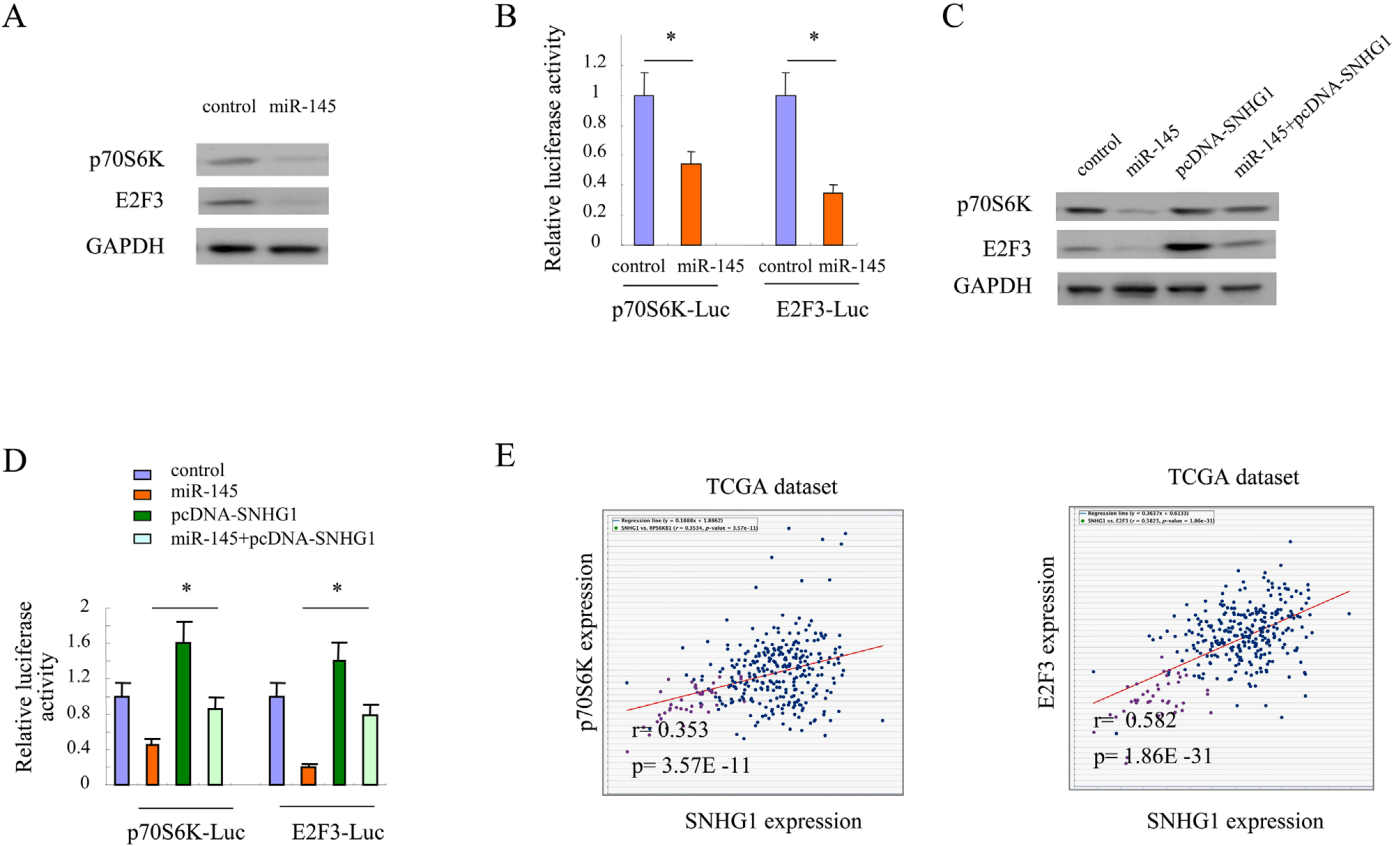
Target genes of miR-145 were modulated by SNHG1 in colorectal cancer cells **(A)** The expression of p70S6K and E2F3 after miR-145 expression in LOVO cells. **(B)** p70S6K-Luc and E2F3-Luc were transfected in LOVO cells in the presence of miR-145 or vector control for 24 h and then luciferase activity was determined. **(C)** The expression of p70S6K and E2F3 after miR-145 or / and SNHG1 expression in LOVO cells. **(D)** p70S6K-Luc and E2F3-Luc were transfected in LOVO cells in the presence of miR-145 or / and SNHG1 for 24 h and then luciferase activity was determined. **(E)** Co-expression of SNHG1 and p70S6K or E2F3 was analyzed through querying open database ChIPBase v2.0 in TCGA colorectal cancer datasets (SNHG1 and p70S6k: r: 0.353, *p*= 3.57E -11; E2F3: r: 0.582, *p*= 1.86E -31). All experiments were performed in triplicate; bars, s.e.m.; ^*^, *p*<0.05.

Previous studies showed that p70S6k and E2F3 were two main targets of miR-145 [34, 35]. miR-145 could suppress tumor progression by inhibiting these two genes. As shown in Figure 4A, overexpression of miR-145 resulted in downregulation of p70S6k and E2F3 in colorectal cancer cells. The luciferase assay also indicated that miR-145 suppressed the expression of these two genes in colorectal cancer cells (^*^, *p<*0.05, Figure 4B). Furthermore, we addressed whether target genes of miR-145 could be regulated by SNHG1 in colorectal cancer cells. As shown in Figure 4C, miR-145 suppressed the expression of p70S6k and E2F3; however, this suppression effect was compromised by overexpression of SNHG1 in LOVO cells. Similarly, the luciferase assay also indicated that the suppression effect of miR-145 on p70S6k and E2F3 were rescued by SNHG1 (^*^, *p<*0.05, Figure 4D). Moreover, co-expression of SNHG1 with either p70S6k or E2F3 was analyzed through querying open database ChIPBase v2.0 in TCGA colorectal cancer datasets [36]. The results showed that SNHG1 was positively correlated with target genes of miR-145 in colorectal cancers (SNHG1 and p70S6k: r: 0.353, *p*= 3.57E -11; E2F3: r: 0.582, *p*= 1.86E -31; Figure 4E). These data suggested that target genes of miR-145 could be regulated by SNHG1 in colorectal cancer cells.

To determine whether the effect of SNHG1-mediated tumor cell proliferation via regulation of miR-145 is dependent on Dicer, we compared the effect of SNHG1 on the cell proliferation in parental HCT116 or HCT116 Dicer^-/-^ cells. As shown in Supplementary Figure 1, in comparison with vector control, SNHG1 enhanced the cell proliferation in HCT116 parental cells (^*^, *p*<0.05; Supplementary Figure 1A). However, the effect of SNHG1 on cell proliferation was attenuated in HCT116 Dicer^-/-^ cells (Supplementary Figure 1B). Similarly, as compared with vector control, the average change in p70S6K or E2F3 expression was less significant in HCT116 Dicer^-/-^ cells. It indicated that the de-repression of p70S6K (Supplementary Figure 1C) or E2F3 (Supplementary Figure 1D) abundance by SNHG1 overexpression was blunted in HCT116 Dicer^-/-^ cells. These results supported the notion that the SNHG1 requires mature miRNAs for its function towards p70S6K or E2F3.

### SNHG1 promotes cell proliferation via suppression of miR-145 in colorectal cancer

**Figure 5:**
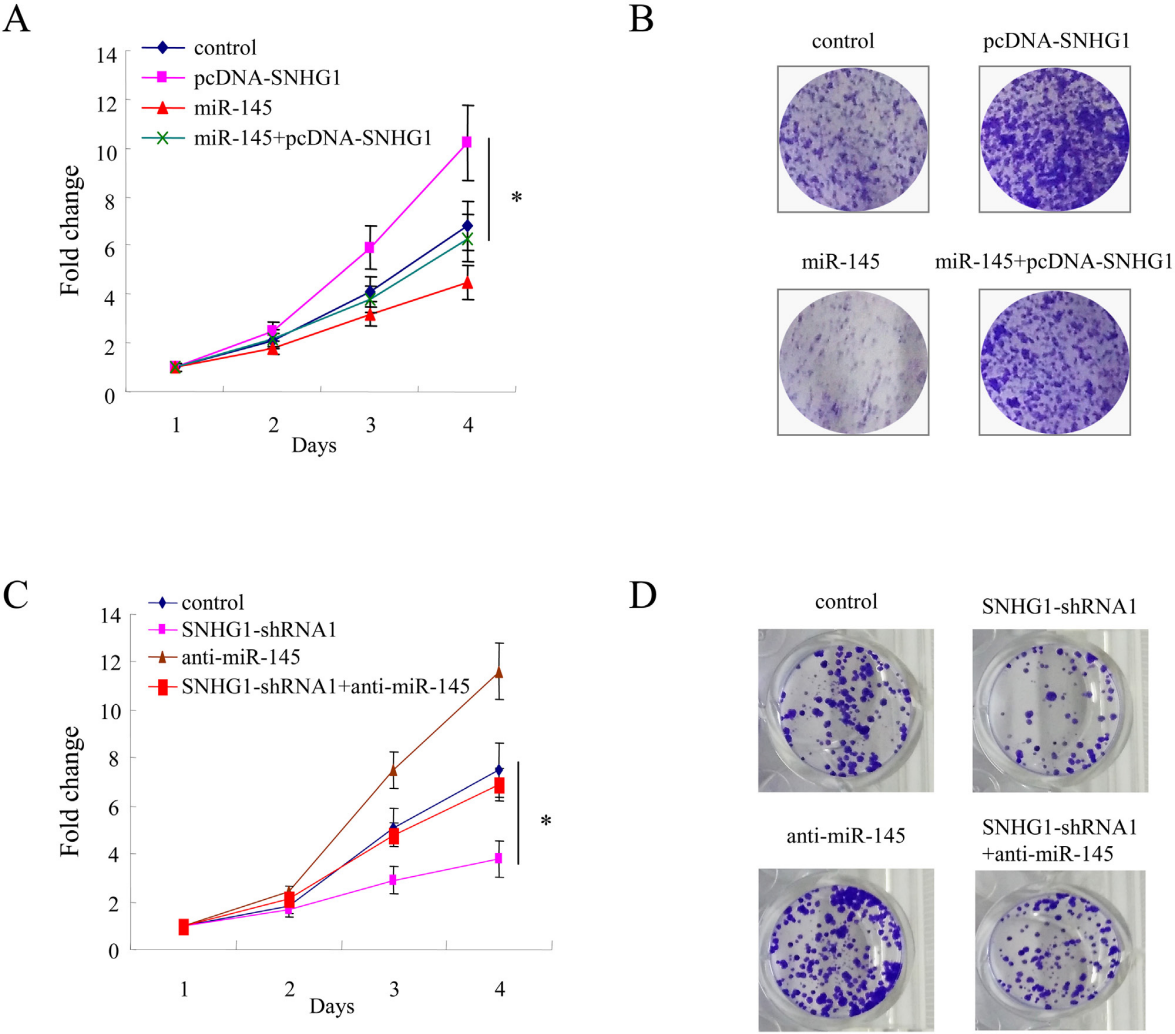
SNHG1 promotes cell proliferation via suppression of miR-145 in colorectal cancer **(A, B)** LOVO cells were overexpressed with SNHG1 or / and miR-145, and then cell proliferation were determined by CCK-8 cell growth assay (A) and colony formation assay (B). **(C, D)** CCK-8 cell growth assay (C) and colony formation assay (D) in LOVO cells of expression of control and SNHG1-shRNA1 with or without anti-miR-145. All experiments were performed in triplicate; bars, s.e.m.; ^*^, *p*<0.05.

We determined whether SNHG1 promoted cell proliferation by repression of miR-145 in colorectal cancer cells. To this end, LOVO cells were overexpressed with SNHG1 in combination with miR-145. As shown in Figure 5A, overexpression of SNHG1 enhanced cell proliferation significantly; however, this effect was partly comprised by introduction of miR-145 in colorectal cells (^*^, *p<*0.05). The colony formation assay also showed miR-145 could attenuate the tumor growth effect conferred by SNHG1 (^*^, *p<*0.05, Figure 5B).

Next, the endogenous SNHG1 was knocked down by specific shRNAs. After knockdown of SNHG1, cell growth were inhibited; however, this cell growth suppression effect conferred by loss of SNHG1 could be rescued by inhibition of endogenous miR-145 in colorectal cancer cells (^*^, *p<*0.05, Figure 5C). Similarly, colony formation assay confirmed the findings from the cell growth assay (^*^, *p<*0.05, Figure 5D). Collectively, these results suggested that SNHG1 promotes cell proliferation by suppression of miR-145 in colorectal cancer cells.

### SNHG1 predicts poor prognosis in colorectal cancer

**Figure 6:**
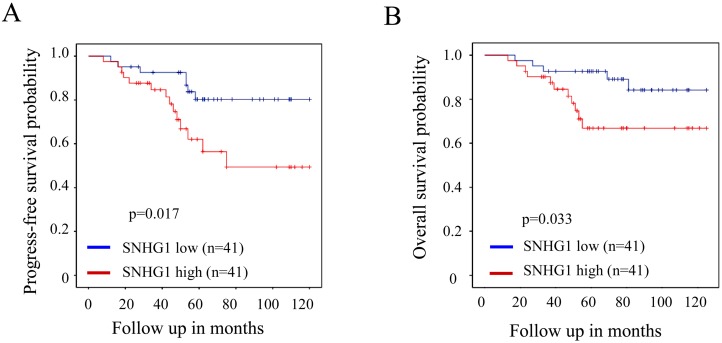
SNHG1 predicts poor prognosis in colorectal cancer **(A, B)** The univariate analysis of progress-free survival (n=82; *p*=0.017) and overall survival (n=82; *p*=0.033) was performed using the Kaplan-Meier analysis in colorectal cancer patient cohort.

Lastly, we determined the relationship between SNHG1 expression and prognosis in patients with colorectal cancer. The univariate analysis of survival was performed using the Kaplan-Meier analysis. As shown in Figure 6A, the results showed that patients with higher SNHG1 expression (n=41) had a much worse progress-free survival than these patients with lower expression of SNHG1 (n=41). The estimated five-year progression-free survival rates for subjects from the high expression and low expression groups were 56.4% and 80.3%, respectively (*P*=0.017). Moreover, the overall survival analysis revealed that patients with higher SNHG1 expression (n=41) had a worse overall survival than these patients with lower expression of SNHG1 (n=41). The estimated five-year overall survival rates for subjects from the high expression and low expression groups were 66.8% and 92.7%, respectively (*p*=0.033; Figure 6B). Collectively, these results suggested that higher expression of SNHG1 predicts poor prognosis in colorectal cancer.

## DISCUSSION

SNHG1 was reported to be involved in the tumorigenesis and progression of several cancers recently [27, 37–42]. It's upregulated in hepatocellular carcinoma, and which may contribute to the tumorigenesis of hepatocellular carcinoma by inhibition of miR-195 [26]. It's also found that SNHG1 could promote cancer progression via inhibition of miR-101-3p and activation of Wnt/β-catenin signaling pathway in lung cancer [28]. Additionally, SNHG1 was reported to act as a sponge for miR-338 to enhance esophageal carcinoma cell growth [30]. In consistent with these findings, our data showed that SNHG1 was overexpressed in colorectal cancers. The expression of SNHG1 was associated with advanced stage and tumor recurrence of colorectal cancers. Furthermore, functional studies revealed that SNHG1 could promote cell growth of colorectal cancer cells both *in vitro* and *in vivo*. Therefore, these results suggested SNHG1 may have an oncogenic role in the development and progression of colorectal cancer.

miRNA-145 (miR-145) is a tumor-suppressive microRNA that participates in the malignant progression of colorectal cancer [34, 35, 43, 44]. For example, epigenetically regulated miR-145 suppresses colon cancer invasion and metastasis by targeting LASP1 [45, 46]. miR-145 inhibits angiogenesis through modulation of connexin-43 expression [47, 48]. Overexpression of miR-145 increases cetuximab-mediated antibody-dependent cellular cytotoxicity in human colon cancer cells [49]. Moreover, miR-145 suppresses cell migration and invasion by targeting paxillin in human colorectal cancer cells [50]. In the present study, our date suggested that the SNHG1 and miR-145 were negatively correlated with each other. Moreover, the expression of SNHG1 and miR-145 could be repressed reciprocally by each other. We also found SNHG1 promoted cell proliferation via suppression of miR-145 in colorectal cancer.

ncRNAs have important roles in the tumor development and progression, and they may function in gene regulatory networks as signals, decoys or scaffold [51, 52]. lncRNA was reported to be a sponge for regulating the expression and activity of miRNA [53]. For example, linc-MD1 sponges miR-133 and miR-135 to regulate the expression of MAML1 and MEF2C to activate muscle-specific gene expression [54]. lincRNA-RoR regulates Oct4, Nanog, and Sox2 in human embryonic stem cell self-renewal by acting as endogenous miRNA sponge [55]. Snhg1 promotes expression of proto-oncogene CST3 by acting as a sponge for miR-338 in primary esophageal cancer cells [30]. In consistent with previous studies, our data revealed that SNHG1 promotes cell proliferation by acting as a sponge of miR-145, and as a result, target genes of miR-145 were modulated by SNHG1 in colorectal cancer.

In conclusion, our study found that SNHG1 was overexpressed in colorectal cancer and promotes cell proliferation by acting as a sponge of miR-145 in colorectal cancer cells. SNHG1 expression was correlated with advanced colorectal cancer stage and tumor recurrence. Moreover, the survival analysis indicated that colorectal cancer patients with higher expression of SNHG1 had a worse prognosis. Therefore, these findings suggested that SNHG1 may act as a potential therapeutic target for the treatment of colorectal cancer.

## MATERIALS AND METHODS

### Cell culture

LOVO and HCT116 cells were purchased from Cell bank of Chinese Academy of Sciences (Shanghai, China). Dicer-deficit HCT116 cells (Dicer^−/−^) was from Center laboratory of the Peking Union Medical College Hospital (PUMCH). They were cultured in Dulbecco's modified Eagle's medium (Hyclone, Logan, UT, USA) supplemented with 10% fetal bovine serum (Hyclone, Logan, UT, USA), 0.1 mg/ml streptomycin, and 100 units/ml penicillin (Invitrogen, California, USA) in 5% CO_2_ atmosphere at 37°C.

### Clinical samples

Colorectal cancer samples were collected at the time of diagnosis from the First Affiliated Hospital of Zhengzhou University. This study was approved by the Research Ethics Committee of Zhengzhou University. Written informed consents were obtained from all patients who provided samples.

### Establishment of stable cell lines

The method of Establishment of stable cell lines were described as previously described. Briefly, lentiviral plasmids expressing SNHG1 or SNHG1-shRNAs were co-transfected with pHelper plasmids in 293T cells. Lentiviral particles were harvested purified with ultracentrifugation from the media after 48 hours of transfection. Cells were then infected with lentiviruses encoding SNHG1 or SNHG1-shRNAs. The efficiency of knockdown was evaluated by real-time PCR.

### Quantitative real-time PCR

Total RNA was isolated by RNeasy mini kit (Qiagen, Germany). cDNA was prepared using the SuperScript® III First-Strand Synthesis System (Invitrogen, California, USA). Quantitative real-time PCR was performed using SYBR Green dye on an Applied Biosystems 7300 Real-time PCR system (Applied Biosystems, Foster City, CA).

### Cell growth assay

Cell Counting Kit-8 (CCK-8, Dojindo, Tokyo, Japan) were used to test the cell proliferation according to the manufacturer's instructions. The absorbance value for each well was measured at 450 nm with a Multiskan FC microplate reader (Thermo scientific, Waltham, MA, USA). Each experiment was conducted three times.

### Colony formation assay

Colony formation assay were described as previously described. Cells (1.0 × 10^3^) were seeded into 24-well plates in 1 ml of complete growth medium. The medium was changed every three days. One to two weeks later, cells were stained by 0.1% crystal violet (Sigma-Aldrich, St. Louis, MO, USA) in methanol for 10 min. Colonies (more than 50μm diameter) were counted directly on the plate.

### Western blot analysis

Western blot analysis was performed as previously described. Briefly, cells were lysed in cold lysis buffer, proteins (20-30μg) were resolved on SDS-PAGE, transferred onto PVDF membranes, and probed with antibodies for p70S6K (14485-1-AP, Proteintech), E2F3 (27615-1-AP, Proteintech) and GAPDH (sc-32233, Santa Cruz Biotechnology) at 4°C overnight. Detection was performed with the SuperSignal West Femto Maximum Sensitivity Substrate Trial Kit (Pierce, Rockford, IL, USA). Detection was carried out with the SuperSignal West Femto Maximum Sensitivity Substrate Trial Kit (Pierce, Rockford, IL, USA). The band images were digitally captured and quantified with a FluorChem FC2 imaging system (Alpha Innotech, San Leandro, CA, USA).

### Gene reporter assays

Gene reporter assays were performed as previously reported. Cells were co-transfected with synthetic miR-145, the wild-type or mutant SNHG1 luciferase reporter vector (SNHG1-WT Luc or SNHG1-MUT Luc) and pRL vector coding for the Renilla luciferase (Promega, Madison, WI, USA), and Cells were then cultured for 24 hours. After that time, cells were collected and luciferase activities were measured using the Dual Luciferase Reporter Assay System (Promega, Madison, WI, USA), according to the manufacturer's instructions.

### Xenograft tumor formation

Mouse xenograft assay were performed as previously described. Briefly, the BALB/c (6-week old) athymic nude mice were purchased from Vital River Laboratory Animal Technology (Charles River Laboratories, Beijing, China). The mice were injected subcutaneously in the flank regions with 2.0 × 10^6^ cells in 0.1 mL of PBS. The tumor size was measured every week with calipers. Four weeks after implantation, mice were euthanized by asphyxiation in a CO_2_ chamber and tumors were excised and examined. All procedures were conducted in accordance to Animal Care and Use Committee guidelines of Zhengzhou University.

### Analysis of microarray data

Oncomine cancer microarray database (http://www.oncomine.org) and TCGA database was used to study gene expression of SNHG1 in colorectal cancer samples as we previously described. Gene expression data were also obtained from NCBI Gene Expression Omnibus (GEO) database (accession numbers: GSE20842), and expression data for SNHG1 were log transformed, median centered per array, and the standard deviation was normalized to one per array. The co-expression analysis of SNHG1 and miR-145 (p70S6K or E2F3) were performed using ChIPBase v2.0 databse.

### Statistical analysis

All data were expressed as mean ±standard error of the mean (SEM). Between groups and among groups comparisons were conducted with Student t test and ANOVA, respectively. Mann-Whitney U test is used for nonparametric variables. The association of SNHG1 expression and clinical characteristics was analyzed by Chi-square or Fisher's two-tailed exact test. Statistical analysis was performed using GraphPad Prism software version 4.0 (PRISM4) (GraphPad Software Inc, LaJolla, CA), and *p*<0.05 was considered significant.

## SUPPLEMENTARY MATERIALS FIGURE


