# Supplementary material for: SNHG1 promotes cell proliferation by acting as a sponge of miR-145 in colorectal cancer
Source: Oncotarget. 2017 Dec 14;9(2):2128–39. doi: 10.18632/oncotarget.23255 (PMC5788627; doi:10.18632/oncotarget.23255)
Supplement: Supplementary file 1 [file oncotarget-09-2128-s001.pdf]

SNHG1 promotes cell proliferation by acting as a sponge of miR-145 in colorectal cancer

SUPPLEMENTARY MATERIALS

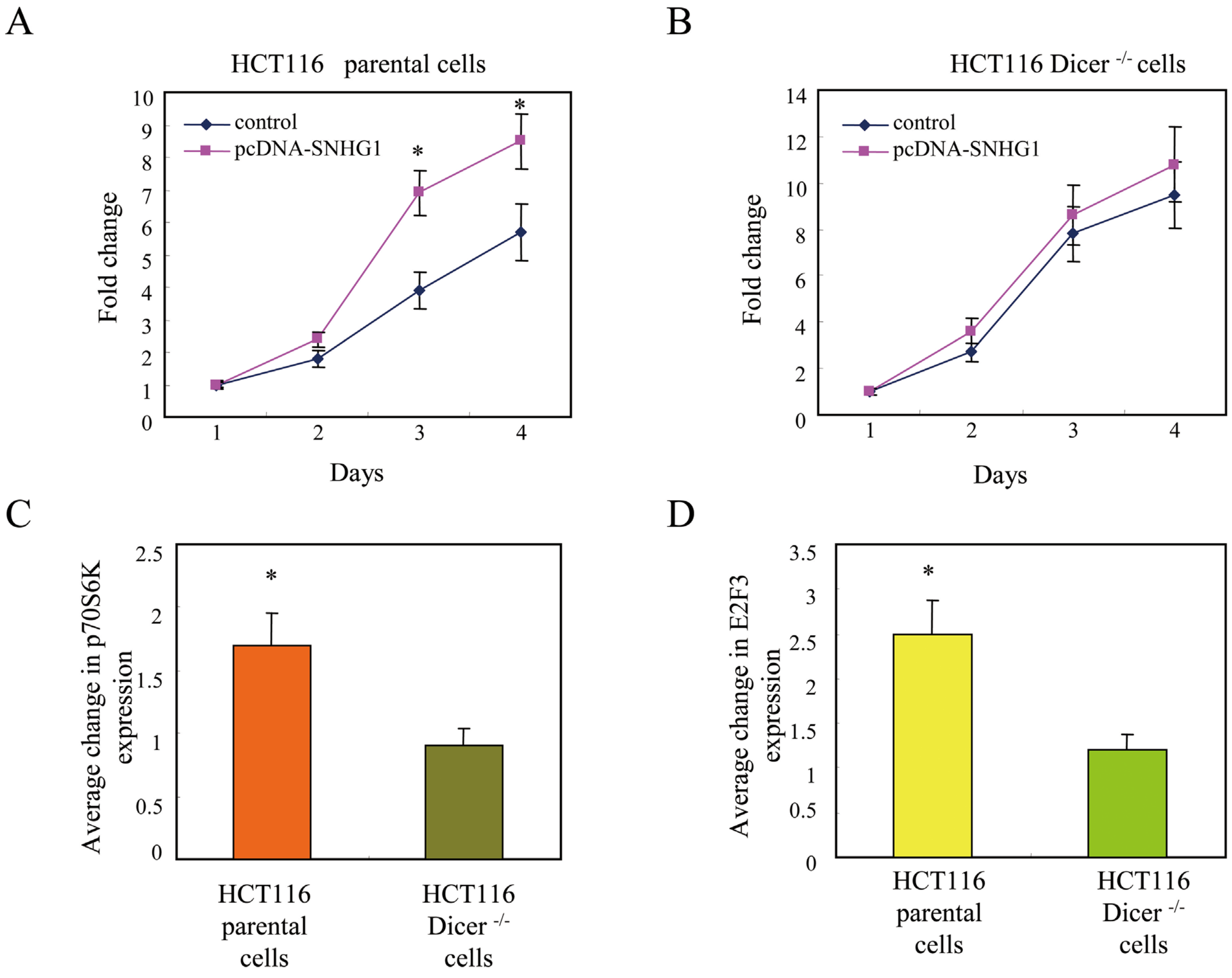

Supplementary Figure 1: SNHG1 requires mature miRNAs for its function towards p70S6K or E2F3. **(A, B)** CCK-8 cell growth assay in parental HCT116 (A) or HCT116 Dicer$^{-/-}$ cells (B) after expression of SNHG1. **(C, D)** p70S6K or E2F3 mRNA levels 24 h after the transfection of pcDNA-SNHG1 in parental HCT116 or HCT116 Dicer$^{-/-}$ cells. Data are normalized using pcDNA vector control-transfected cells. All experiments were performed in triplicate; bars, s.e.m.; *, $p<0.05$.
